# The Contradictory Effects of Neuronal Hyperexcitation on Adult Hippocampal Neurogenesis

**DOI:** 10.3389/fnins.2016.00074

**Published:** 2016-03-03

**Authors:** José R. Pineda, Juan M. Encinas

**Affiliations:** ^1^Laboratory of Neural Stem Cells and Neurogenesis, Achucarro Basque Center for NeuroscienceZamudio, Spain; ^2^IKERBASQUE, The Basque Foundation for ScienceBilbao, Spain

**Keywords:** neurogenesis, hippocampus, neural stem cells, hyperexcitation, epilepsy

## Abstract

Adult hippocampal neurogenesis is a highly plastic process that responds swiftly to neuronal activity. Adult hippocampal neurogenesis can be regulated at the level of neural stem cell recruitment and activation, progenitor proliferation, as well as newborn cell survival and differentiation. An “excitation-neurogenesis” rule was proposed after the demonstration of the capability of cultured neural stem and progenitor cells to intrinsically sense neuronal excitatory activity. *In vivo*, this property has remained elusive although recently the direct response of neural stem cells to GABA in the hippocampus via GABA_A_ receptors has evidenced a mechanism for a direct talk between neurons and neural stem cells. As it is pro-neurogenic, the effect of excitatory neuronal activity has been generally considered beneficial. But what happens in situations of neuronal hyperactivity in which neurogenesis can be dramatically boosted? In animal models, electroconvulsive shock markedly increases neurogenesis. On the contrary, in epilepsy rodent models, seizures induce the generation of misplaced neurons with abnormal morphological and electrophysiological properties, namely aberrant neurogenesis. We will herein discuss what is known about the mechanisms of influence of neurons on neural stem cells, as well as the severe effects of neuronal hyperexcitation on hippocampal neurogenesis.

## Introduction

Neurogenesis in the hippocampus starts with the activation of quiescent neural stem cells (NSCs), the first regulatory step that will determine the amount of new neurons generated in a given time point. Although neurogenesis is tightly linked to the level of hippocampal circuitry activity it has only been recently unveiled how gamma-aminobutyric acid (GABA) plays an essential role into translating neuronal activity into NSCs activation, as we will explain below. The question that we want to address in this review is what occurs when neuronal activity is increased to abnormal hyperexcitatory levels, especially in the clinically relevant context of epilepsy or electroconvulsive therapy (ECT) and its animal model, electroconvulsive shock (ECS). Two aspects make this question important. First, the possibility that enhanced activation of NSCs leads to a premature exhaustion of the NSC population and of neurogenesis; and second the existence of aberrant neurogenesis, i.e., the generation of neurons with ectopic location and different morphological and electrophysiological properties that can contribute to epilepsy.

NSCs, in rodents, have been shown to be able to divide symmetrically to generate more copies of themselves (Bonaguidi et al., 2011), and therefore the possibility exist that they can expand their pool. However, in normal conditions, this capability does not counteract the depletion of the NSC population that associates with age (Encinas and Enikolopov, 2008; Bonaguidi et al., 2011; Walter et al., 2011; Andersen et al., 2014). The depletion of the population is based on the activation-coupled astrocytic differentiation of NSCs. Most of the NSCs remain quiescent but once they get activated they undergo a round of several asymmetric divisions to generate neuronal precursors that either die by apoptosis and are removed by microglia, or become mature granule cells (Sierra et al., 2010). At least in mice, after finishing their round of asymmetric cell divisions NSCs differentiate into astrocytes losing their stem cell capabilities (Encinas et al., 2011). Thus, the level of activation of NSCs not only determines the level of neurogenesis but also the rate of depletion of NSCs. The prediction derived from this model is that increased activation of NSCs would lead to an initial boost of neurogenesis followed by diminished neurogenesis in the long term. This model would explain the seemingly contradictory results regarding hippocampal cell proliferation and neurogenesis in human epilepsy. Noteworthy, the dynamics of neurogenesis decline in normal conditions in humans might be different as pointed out by carbon-14-based methods (Spalding et al., 2013).

Several *in vivo* manipulations or brain alterations that influence electrical activity also affect adult neurogenesis. Seizures trigger an early increase of cell proliferation that involved NSCs (Huttmann et al., 2003; Indulekha et al., 2010). Also, ECS has been consistently reported (Segi-Nishida et al., 2008; Jun et al., 2015) to increase NSC recruitment and activation of NSCs. However, long-term studies addressing the fate of dividing NSCs as well as the chronic effect on the neurogenic niche are still missing. Both models of hyperexcitation are usually accompanied with the induction of neuronal death in granule cells (Zarubenko et al., 2005; Dingledine et al., 2014), which in turn might affect neurogenesis. We will focus on two aspects of neurogenesis that seem to be key regarding neuronal hyperexcitation in the hippocampus, activation and exhaustion of NSCs, and the induction of aberrant neurogenesis.

## Activation and exhaustion of NSCs

We have recently shown that in mice seizures trigger a dramatic response in the dentate gyrus (DG) leading to a swift and almost complete disruption of the neurogenic niche (Sierra et al., 2015). The main effect of seizures is to activate NSCs and induce them to differentiate into reactive astrocytes. Thus, the neurogenic potential is abandoned and the NSC pool rapidly depletes. Even though seizures trigger an initial boost of cell proliferation, mainly accounted for the activation of NSCs, in following weeks cell proliferation and neurogenesis diminish to a minimum (Sierra et al., 2015). These results could explain the chronic impairment observed in two rat models of temporal lobe epilepsy (TLE; Hattiangady et al., 2004; Hattiangady and Shetty, 2010). They also are in agreement with part of the data obtained from human samples and provide and explanation for them. Because mesial TLE (MTLE) is often resistant to drugs, surgical resection of the hippocampus as a last-resort therapeutic strategy to stop seizures is frequently performed. These samples represent a valuable source of tissue that can be analyzed without any of the drawbacks of postmortem tissue, such as degradation and overfixation.

Nestin-positive radial cells considered as putative NCSs were not found in samples from adult MTLE patients, leading the authors to suggest that the neural stem/progenitor pool might be depleted by chronic seizure activity in humans (Blümcke et al., 2001), a hypothesis that is supported in rodents by studies showing how seizure indeed recruit and activate NSCs in significantly manner (Huttmann et al., 2003) that later translates into an almost total depletion of the NSC pool (Sierra et al., 2015). Furthermore, e*x vivo* analysis of hippocampal neurogenesis showed that even though the epileptic human hippocampus could contain neural progenitors (Blümcke et al., 2001), these were absent in epilepsy patients with hippocampal sclerosis (Paradisi et al., 2010). The data obtained, however, are not consistent and sometimes are even contradictory when measuring other parameters. Using these samples from intractable-MTLE patients it has been shown that the immunoreactivity for PSA-NCAM (polysialic acid neural cell adhesion molecule) a specific marker of neuroblasts, or immature neurons, was lost in the neurogenic niche of the dentate gyrus (Mikkonen et al., 1998). A similar result was reported later, describing how the frequency and early onset of seizures correlated with decreased adult neurogenesis (Mathern et al., 2002). The mRNA expression of another marker of neuroblasts, doublecortin (DCX), was also decreased in MTLE patients compared to age-matched controls (Fahrner et al., 2007). The protein levels, however, did not change significantly. In contrast, an increase in the number of neural progenitors in MTLE has been suggested based on the expression of Musashi, a stem cell marker functionally related to self-renewal (Crespel et al., 2005). However, the precise cell type expressing Musashi, as well as the function of this protein in the adult hippocampus has not been explored. More conflicting evidence regarding proliferation in the MTLE hippocampus has been reported. For instance, no significant change in proliferation was found by labeling with Ki67 or minichromosome 2 (mcm2; Fahrner et al., 2007), a DNA replication licensing protein; although another study reported increased proliferation employing mcm2 (Thom et al., 2005). In general it might be inferred that an initial wave of proliferation could be followed by diminished cell division and neurogenesis, and that the age of onset, frequency and severity of the seizures will determine the neurogenic outcome in the long run. It must be considered also that human samples are obtained from individuals resistant to pharmacological treatment, which might represent only a particular subset of patients of MTLE.

### Aberrant neurogenesis

One of the most common findings in experimental models of MTLE, as well as in human samples is aberrant neurogenesis, i.e., the generation of neurons with ectopic location (located mostly into the hilus but also in the molecular layer), with abnormal ultrastructural (Dashtipour et al., 2001) and morphological features including network reorganization through mossy fiber sprouting (Parent et al., 1997), and with altered electrophysiological properties (Scharfman, 2000; Scharfman et al., 2003). One explanation for the ectopic location of neurons in MTLE could be the loss of reelin. Seizures cause death of the reelin-expressing interneurons that populate the hilus; PSA-NCAM neuroblasts express the downstream reelin signaling molecule Dab1; and *in vitro*, the migration of neuronal precursors is altered by manipulating the levels of reelin (Gong et al., 2007). The involvement of reelin could explain the existence of different levels of aberrant neurogenesis even when neurogenesis is greatly impaired (Murphy et al., 2012; Sierra et al., 2015). It has also been shown, that in mice lacking fibroblast growth factor (FGF) 22, ectopic location of newborn neurons is significantly reduced, suggestion that FGF22 might be playing a role in seizure-induced abnormal migration of neuroblasts (Lee and Umemori, 2013). Newly-born and developing granule cells are more sensitive to seizures than the mature and preexistent ones, and undergo noticeable changes such as the abnormal development of basal dendrites (Walter et al., 2007). The contribution of aberrant neurogenesis to MTLE is still not fully understood. A positive correlation between the number of ectopic newborn granule cells, mossy fiber sprouting, and loss of mossy cells; and the frequency of seizures was found in the intraperitoneal-pilocarpine model of mouse epilepsy (Hester and Danzer, 2013). No conclusions on causality can be extracted form that study. However, it has been recently shown that reducing neurogenesis by triggering apoptosis in dividing nestin-expressing cell reduced aberrant neurogenesis and lead to a reduction of the frequency of chronic seizures, but not of the severity or duration (Cho et al., 2015). This positive effect could not be attributed only to decreased aberrant neurogenesis, as “normal” neurogenesis was reduced as well. Importantly, the positive effect on the frequency of spontaneous seizures was abolished when the generation of reactive astrocytes following the induction of seizures was impaired.

## Potential mechanisms

### GABA signaling

Confirming previous results obtained *in vitro*, showing that GABA_A_ receptors are expressed in neural progenitors *in vitro* (Farrant and Nusser, 2005; Ge et al., 2007), it has been more recently reported how NSCs respond directly to GABA via GABA_A_ and GABA_B_ receptors (Song et al., 2012; Giachino et al., 2014). GABA released by paravalbumin-expressing interneurons acts tonically on NSCs maintaining them in quiescence. Administration of the GABA_A_ receptor agonist muscimol reduces the number of NSCs that enter the cell cycle whereas knocking down of the γ2 subunit of the GABA_A_ receptor induces the activation of a higher number of NSCs. Interestingly, blocking the action of GABA in NSCs not only increases activation but also promotes symmetrical cell division (Song et al., 2012). In the context of MTLE, and assuming that the results found in mice regarding the massive activation and loss of NSCs (Sierra et al., 2015) could be similar in humans, treatments based on activating GABA_A_ receptors (such as benzodiazepines) could be directly beneficial as they would preserve neurogenesis by promoting quiescence, and therefore preserving the NSC population. In a similar fashion, both the knock-out of the GABA_B1_ receptor and the infusion of its antagonist CGP54626A increased NSC activation, although in this case an expansion of the NSCs population, expected if symmetric division was augmented, was not found. Administration of the GABA_B1_ receptor baclofen decreased the number of NSCs in division (Giachino et al., 2014). Interestingly neuroblasts are a major source of GABA suggesting a retro-control or feedback mechanism for NSC quiescence as GABA exerts a tonic inhibitory control of NSC proliferation (Liu et al., 2005). In the SVZ, it was shown *in vivo* that treatments with the GABA_A_R agonist muscimol decreased cell proliferation and the number of label-retaining stem cells (LRSCs), whereas the blockade of GABA_A_R signaling with the specific inhibitor bicuculline increased mitosis and the number of LRSCs (Fernando et al., 2011). The authors concluded that the inhibitor bicuculline primarily increased the rate of division of already cycling stem cells. However, more recent data in which cycling cells were eradicated by using exposure to γ-radiation, showed that muscimol or bicuculline delayed and increased (respectively) the entry of quiescent NSCs into the cell cycle (Daynac et al., 2013). In the hippocampus, during the progression of the MTLE alterations of the GABAergic neuronal circuitry also take place (Maru and Ura, 2014), which in turn could affect directly NSCs. Interestingly tonic GABAergic signaling from PV can prevent their proliferation and subsequent maturation or return them to quiescence if previously activated (Moss and Toni, 2013; Song et al., 2013). Moreover, PV interneurons are capable of suppress neurogenesis during periods of high network activity and, on the other hand, facilitate neurogenesis when network activity is low (Song et al., 2012). The efficacy of GABAergic synaptic inhibition is a principal factor in controlling neuronal activity. Recent studies demonstrated that GABA_A_-based synaptic inhibition is decreased in the hippocampal CA1 area of patients with intractable MTLE (Maru and Ura, 2014). It remains to be elucidated what happens in local PV circuitry during seizures and if stimulation of PV in this context could be a therapeutic tool to control NSC massive activation.

Finally, another manner in which GABAergic interneurons regulate excitability is through direct action of a 36-amino acid peptide called neuropeptide Y (NPY; Colmers and El Bahh, 2003) and norepinephrine (NE; Jhaveri et al., 2015). Both are potent endogenous anticonvulsants (Erickson et al., 1996; Baraban et al., 1997; Woldbye et al., 1997; Szot et al., 1999; Weinshenker et al., 2001). Gene expression of NPY has been found to be upregulated in the hippocampus either after induction of seizures or ECS (Gruber et al., 1994; Kragh et al., 1994). It has been speculated that both transmitters, NPY and NE, likely dampen excessive excitation of neurons in brain regions implicated in epileptic seizures. However, recent findings have been demonstrated that both peptides are able to independently promote proliferation of hippocampal neural stem and progenitor cells (Decressac et al., 2011; Jhaveri et al., 2015). These findings propose that aberrant neural activity is a master key to provoke deregulation of the fine-tuning control of NSC activation and progenitor proliferation. Therefore, GABAergic input seems to be a key regulator of NSCs activation and neurogenesis, as it also has effects on other steps of the neurogenic cascade, namely survival and differentiation of neuronal progenitors (Ge et al., 2007; Song et al., 2012; Giachino et al., 2014). However, other regulatory pathways might exist and unveiling their interplay will provide the ultimate understanding of NSC activation in physiological conditions.

### Other mechanisms

Up to date it was believed that mitogenic factors participating on the induction of neurogenesis were released by dying neurons and reactive glia. In severe epilepsy such as MTLE the progression of the disease leads to a severe neuronal loss in the hippocampus (Dericioglu et al., 2013). The release of mitogenic factors can be, however, faster. After generation of seizures Shh protein from Hedgehog signaling pathway, growth factors such as FGF-2, neurotrophins such as BDNF were found to be acutely upregulated in hippocampal tissue before there was neuronal loss and then progressively diminished in chronic epilepsy (Riva et al., 1992; Lowenstein et al., 1993; Gall et al., 1994; Shetty et al., 2003, 2004; Hattiangady et al., 2004). The majority of these factors were upregulated during acute seizures, potentially reflecting an initial response to neural activity (as it happens also in ECS), independently of neuronal cell death.

### BDNF neurotrophin

Hippocampal network activity stimulates transcription of the Brain-derived neurotrophic factor (BDNF) gene and the translation of *Bdnf* mRNA (Mattson, 2008; Kazanis et al., 2010). BDNF is neuroprotective in a wide variety of brain pathologies (Zeev et al., 2009; Zuccato and Cattaneo, 2009). In the hippocampus BDNF is able to modulate synaptic transmission (Huang and Reichardt, 2001; Waterhouse and Xu, 2009), and in the cortex, it participates in the maturation of GABAergic inhibitory networks (Huang et al., 1999; Hong et al., 2008). Locally synthesized BDNF in dendrites of granule cells promotes differentiation and maturation of progenitor cells in the SGZ by enhancing GABA release from PV GABAergic interneurons (Waterhouse et al., 2012). Several studies have determined that seizure activity is able to increase both mRNA and protein levels (Bengzon et al., 1993). Other studies suggest that an upregulation of BDNF levels could contribute to epileptogenesis (Binder, 2004; Lähteinen et al., 2004), although at the same time it was proposed that its upregulation could be protective for neurons from excitotoxicity (Wu et al., 2004; Pérez-Navarro et al., 2005). Regarding neurogenesis, BDNF is a neurotrophin that promotes proliferation of human fetal neural stem and progenitor cells *in vitro* (Zhang et al., 2011) and it is a potent regulator of the survival and differentiation of adult NSCs (Park and Poo, 2013), suggesting another mechanism linking the effect of hyperexcitotoxycity on stem cell activation and neurogenesis. I has been shown that in glioma-cell populations containing cancer stem cells BDNF is able to increase directly cell division through Akt activation and PTEN inactivation (Tamura et al., 1999; Bertrand et al., 2009). Although Akt and PTEN also has been independently implicated in the proliferation of neural stem and progenitor cells (Amiri et al., 2012; Cai et al., 2014) it remains to be elucidated whether BDNF is the direct regulator.

### Stimulation by FGF

Neuronal activity also can regulate growth factors such as basic FGF(bFGF) and FGF-2 (Riva et al., 1992). FGF-2 overexpression increases excitability and seizure susceptibility (Zucchini et al., 2008) and is acutely overexpressed after seizures (Indulekha et al., 2010). It is well stablished that the morphology of reactive astrocytes is controlled by FGF signaling. In a recent work Goldshmit et al. demonstrated this pleiotropic cytokine is able to decrease gliosis and increase radial glia and neural progenitor cells in spinal cord injury (Goldshmit et al., 2014). However, Kang et al. demonstrated that FGF signaling in brain is responsible for astrocyte hypertrophy in response to an inflammatory stimulus (Kang et al., 2014). Previous studies demonstrated that the expression of FGF-2 and its receptors is induced in astrocytes after epileptiform activity using KA injections in rats (Van Der Wal et al., 1994). FGF signaling is a strong mitogenic factor *in vitro* and *in vivo* when injected subcutaneously or in an intravitreal manner, stimulating cellular proliferation including astrocytes (Lewis et al., 1992; Wagner et al., 1999). In addition, in FGF-2 knock-out mice, intraperitoneal KA injection fails to trigger an increase in cell proliferation, as it does in wild-type mice (Yoshimura et al., 2001). These results support the idea that endogenously synthesized FGF-2 is necessary to stimulate adult hippocampal neurogenesis after brain insult. Interestingly, in the early phase of acute epilepsy FGF-2-expressing reactive astrocytes are observed (Erkanli et al., 2007). However, FGF-2 expression declines considerably in human chronic epilepsy (Hattiangady et al., 2004), with a decrease in parallel of the number FGF-2-positive reactive astrocytes (Erkanli et al., 2007). Reactive astrocytes persist in the chronically epileptic hippocampus, but it remains unclear whether reactive astrocytes that emerge in the early phase after SE persist for prolonged periods of time, or there is turnover and new reactive astrocytes are added progressively. The involvement of FGF signaling in the transformation of NSCs into hypertrophic reactive astrocytes (Sierra et al., 2015) has not yet been addressed.

### Sonic hedgehog signaling

Sonic hedgehog (Shh) signal acts directly on the astrocytes and is sufficient to provoke stem cell response in both models *in vitro* and *in vivo* (Sirko et al., 2013). Shh is one of three ligands for hedegehog (Hh) signaling in mammals (Washington Smoak et al., 2005). When secreted glycoprotein Shh binds Ptc receptor on the cell surface it relieves the inhibition of Smo. Activated Smo goes to the nucleus and triggers the activation of transcription factors, which regulate proliferation, migration, and differentiation. Mice lacking Smo in NSC during development have a small DG with reduced proliferation and reduced generation of neurons (Breunig et al., 2008; Han et al., 2008).

Previous studies of ECS observed a strong and robust increase of Hh signaling through Ptc upregulation and a rapid and reduction of Smo in the hippocampal DG, proposing that both acute and chronic ECS enhanced Shh signaling in the adult hippocampus (Banerjee et al., 2005). In other experiments using the Smo antagonist cyclopamine Lai et al. observed a reduction of hippocampal neural progenitor proliferation *in vivo* (Lai et al., 2003). In agreement with the effects of ECS, synaptic activity involving glutamatergic transmission is proposed to regulate Smo protein, suggesting additional roles for Hh signaling in the control of hippocampal functions (Palma et al., 2005). In the KA model of epilepsy, Shh expression and release by astrocytes induces its own activation in a positive feedback loop, boosting further autocrine Shh release which translates ultimately into increased astrocytes proliferation and conversion into reactive astrocytes (Pitter et al., 2014). One study documented increased expression of Shh by neurons in the epileptic temporal lobe of human and experimental rats, although the consequences of elevated Shh were not studied (Fang et al., 2011).

## Conclusions

Even though the relationship between epilepsy affecting the hippocampus and adult hippocampal neurogenesis has been known for almost two decades, many basic questions remain unsolved. One of the characteristics of the studies published so far is the apparent differential or even contradictory results among them. More than perceiving these controversial results as fruit of inconsistencies due to different animal models and the impossibility to control certain variables (especially when analyzing human tissue), we believe that they are reflecting the overwhelming biological plasticity of neurogenesis. Alterations of neurogenesis in different directions (excessive, aberrant and impaired neurogenesis) in the pathophysiology of epilepsy might be relevant to explain at least some of the cognitive symptoms associated to this disorder and we therefore conclude that further research should be carried out with an open mind in lieu of the variety of possible outcomes.

## Author contributions

All authors listed, have made substantial, direct and intellectual contribution to the work, and approved it for publication.

## Funding

This work was supported by a grant from the Spanish Ministry of Economy and Competitiveness (MINECO) with FEDER funds to JE (SAF2012-40085), and with MINECO Ramón y Cajal contracts to JE (RyC-2012-11185) and JP (RyC-2013-13450).

### Conflict of interest statement

The authors declare that the research was conducted in the absence of any commercial or financial relationships that could be construed as a potential conflict of interest.
